# Tracking disease progression by searching paths in a temporal network of biological processes

**DOI:** 10.1371/journal.pone.0176172

**Published:** 2017-04-27

**Authors:** Rajat Anand, Samrat Chatterjee

**Affiliations:** Computational and Mathematical Biology Lab, Drug Discovery Research Centre, Translational Health Science and Technology Institute, NCR Biotech Science Cluster, Faridabad, India; Universite Libre de Bruxelles, BELGIUM

## Abstract

Metabolic disorders such as obesity and diabetes are diseases which develop gradually over time through the perturbations of biological processes. These perturbed biological processes usually work in an interdependent way. Systematic experiments tracking disease progression at gene level are usually conducted through a temporal microarray data. There is a need for developing methods to analyze such highly complex data to capture disease progression at the molecular level. In the present study, we have considered temporal microarray data from an experiment conducted to study development of obesity and diabetes in mice. We first constructed a network between biological processes through common genes. We analyzed the data to obtain perturbed biological processes at each time point. Finally, we used the biological process network to find links between these perturbed biological processes. This enabled us to identify paths linking initial perturbed processes with final perturbed processes which capture disease progression. Using different datasets and statistical tests, we established that these paths are highly precise to the dataset from which these are obtained. We also established that the connecting genes present in these paths might contain some biological information and thus can be used for further mechanistic studies. The methods developed in our study are also applicable to a broad array of temporal data.

## 1 Introduction

High throughput data like Microarray [1, 2] or RNAseq [3] are used to study systematically a disease condition or how organism is responding to different conditions of the experiment [4]. To study a disease condition from such a high throughput data, instead of looking at expression levels of each gene one by one, it is more informative to look at biological processes perturbed at different experimental conditions [4]. The list of biological processes perturbed in a given experiment can be found by clustering/biclustering the microarray data using relevant algorithms [5, 6]. One can then find biological processes significantly enriched in each cluster using tools such as enrichr [7]. Other methods such as Gene Set Enrichment Analysis [8] finds processes/gene lists which significantly correlate with a phenotype of interest. Methods such as Gene Network Enrichment Analysis [9] finds high transcriptionally affected sub network in a PPI network and looks for significant overlap with a biological process and gives biological processes perturbed at multiple conditions.

Similar methods have been used to study disease condition by identifying significantly perturbed biological processes in different stages in a disease progression. For example, Sun, et al., [10] measured gene expression of different tissues taken from normal and diabetic rats grown for 20 weeks starting from 4 weeks and sacrificed at every 4 weeks. They identified differential expression networks for different time points and found pathways enriched in those networks. In another study, He, et al., [11] looked at molecular mechanisms underlying the hepatocarcinogenesis (HCC) induced by Hepatitis C Virus (HCV) infection. They took the six pathologically defined disease stages in the development and progression of HCC after HCV infection. They found significant functions represented by the progression related sub networks. Tomlins, et al., [12] studied prostate cancer progression by extracting signature genes whose expression significantly correlate with samples representing cancer progression. That is, high/low expression of signature genes in samples representing starting of progression, gradually going to low/high expression in samples representing end of progression. Then, they linked the signature genes with a database of molecular concepts i.e. sets of biologically linked genes. Another study have analysed the temporal respose of human islet cells and rat INS-1E cells to cytokines and inferred a regulatory network from human time expression datasets to select genes for experimental validation [13].

However, as far as we know, no such study has been done to link given such lists of perturbed processes at respective stages in a disease progression. Linking such processes temporally could provide a view of how processes perturbed initially lead to processes perturbed at a later time point in a disease development. Thus, we have used here a temporal microarray data obtained from a mice liver as it was fed on high fat high sucrose diet and progressed towards obese condition. We found the lists of biological processes perturbed at different time points. Then, we constructed a network of biological processes where the processes are connected through common genes. From this network, we have identified links between biological processes that are perturbed at respective time points. These linked biological processes, defined here as paths, connect initial to the final perturbed processes. These paths helped us to study disease progression temporally at a process level and may be used to identify key genes involved in obesity disease progression.

## 2 Results

### 2.1 Gene set enrichment analysis to find perturbed processes

**Fig 1:**
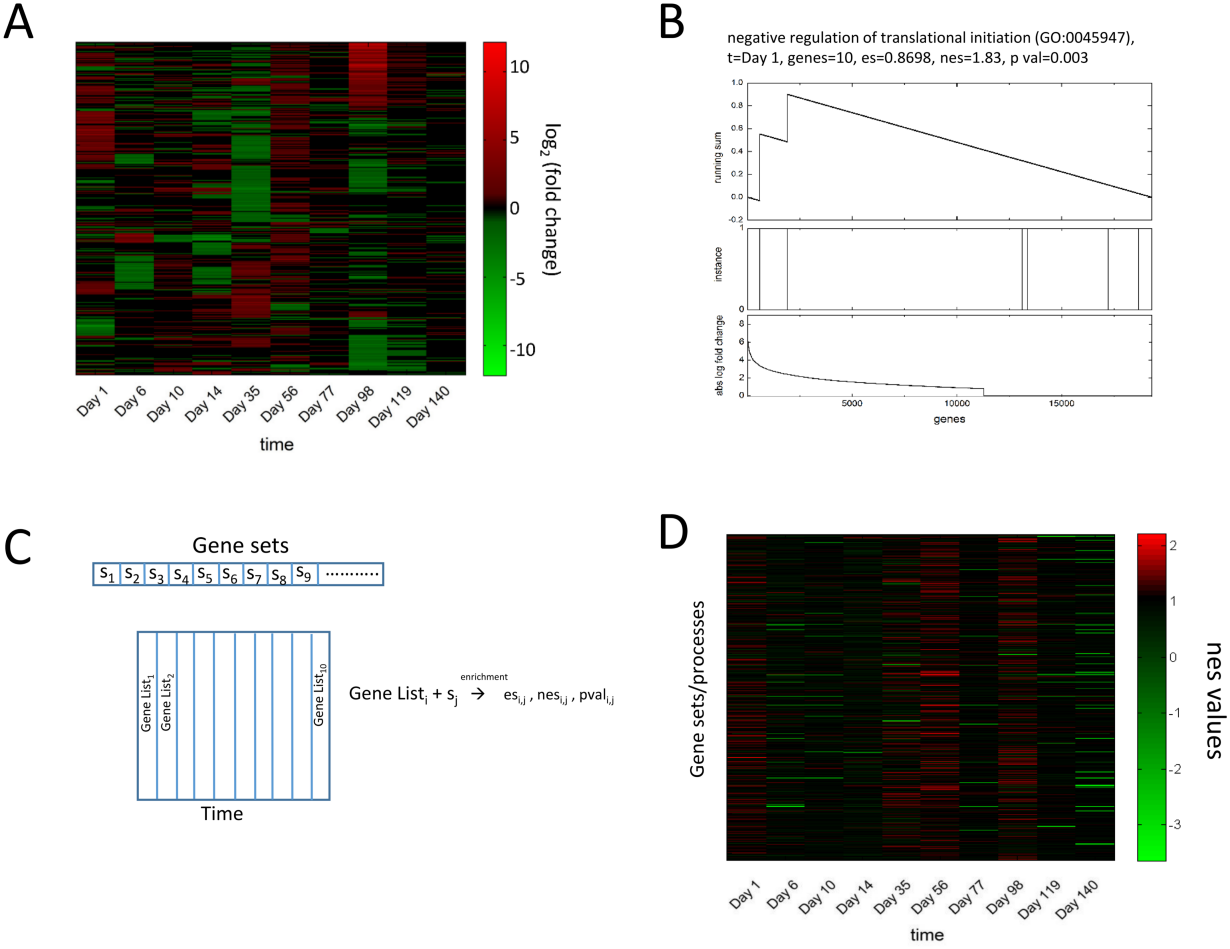
Gene set enrichment analysis to find perturbed biological processes at each time point. (A) Heatmap of the microarray data is shown with x axis as time points, y axis as genes (19303 in total) and color representing fold change (log2 transformed). The heatmap is shown after hierarchical clustering of the data using an algorithm by eisen et al. [5]. (B) An example of gene set enrichment analysis method is shown for a process receiving high nes value at Day 1. The running sum used to calculate es values is shown along with instances of genes of the biological process along the ordered gene list. The ordered gene list is obtained by sorting all the genes according to the absolute log fold change values of genes at Day 1. The absolute log fold change values are also shown. The process contains 10 genes and is highly perturbed at Day 1. (C) Ordering the genes (total 19303) according to fold change values at each time point gives different gene lists for each time point. For each such gene list and each gene set in our database of biological processes, we use the procedure given in (B) to obtain es, nes and pvalues. (D) The nes values obtained using procedure in (B) and (C) for gene lists for each time point and 816 gene sets can be represented in matrix form and is shown as a heatmap.

The details of the experiment conducted and processing of the data is given in Materials and Methods: Processing of Microarray Data. A heat map of the microarray data showing the gene patterns is shown in Fig 1A. To find the processes represented by the genes perturbed at each time point of the experiment, we used gene set enrichment analysis method. For this method, we used a list of biological processes [14] present in enrichr database [7] and processed it (Materials and methods: Gene Ontology Biological Process List) to get in 816 process represented by 10346 genes. In the gene set enrichment analysis, briefly, for each time point, the genes are first sorted by the absolute values of fold change in descending order to get a sorted gene list. Then, it was checked whether the genes belonging to a biological process (a gene set) are significantly present at the top of the list or not by calculating the running sum, the enrichment score (es), the normalized enrichment score (nes) and the pvalue (Materials and methods: Calculation of es, nes and pvalues). For example, a process whose gene set enrichment analysis gave high nes value at Day1, the es value was calculated using the running sum, see Fig 1B top panel. Second panel of Fig 1B depicts the instances where the genes of this set are appearing in this ordered list. The third panel of Fig 1B shows the absolute log fold change values of genes in the ordered list. Most instances of genes of this set are present towards left which results in high es and nes value and signifies that most genes of this process are perturbed. This can be seen more clearly for another process in S2 Fig. We repeated this procedure for each of the 816 biological processes and for each one of the time point (Fig 1C).

This resulted in matrices of es, nes and pvalues. The matrix consisting of nes values of dimension 816 X 10 is shown as a heatmap in Fig 1D. We plotted these nes values for all processes for all time points against their corresponding p values in S3A Fig. The plot shows that processes with high nes values have low p values and so, they are significantly perturbed as expected. Hence, the average absolute fold change of these processes would be high which is confirmed in S3B Fig. This increased our confidence of using high nes value processes in filtering significantly perturbed processes in our analysis. Fig 1D shows that more processes are perturbed in Day 1, Day 35, Day 56 and Day 98 which correspond to large perturbed genes on those time points seen in Fig 1A. To look at actual processes perturbed at each time point, we chose top 5 processes with highest nes values from each time point and show them in Table 1. The table shows that Metabolic and signaling related processes are perturbed in Day 1, then inflammation related processes on Day 6 and Day 10. However, no apparent connection between these processes can be inferred from this table. To address this question, we first constructed a network of biological processes and then overlaid our nes values to capture the links between perturbed biological processes as explained in next section.

**Table 1:** Enriched biological processes.

Time	GO Biological Process (Number of genes, nes value, P-value)	Time	GO Biological Process (Number of genes, nes value, P-value)
**Day 1**	'preassembly of GPI anchor in ER membrane (GO:0016254)' (16, 2.12, 0) 'negative regulation of translational initiation (GO:0045947)' (18, 1.83, 0.003) 'purineribonucleoside bisphosphate metabolic process (GO:0034035)' (17, 1.82, 0.001) '3''-phosphoadenosine 5''-phosphosulfate metabolic process (GO:0050427)' (17, 1.82,0.001) 'hydrogen peroxide catabolic process (GO:0042744)' (20, 1.80, 0.002)	**Day 56**	'termination of RNA polymerase I transcription (GO:0006363)' (24, 2.20, 0.005) 'pseudouridine synthesis (GO:0001522)' (17, 2.12, 0.013) 'bile acid biosynthetic process (GO:0006699)' (21, 2.08, 0) 'SCF-dependent proteasomal ubiquitin-dependent protein catabolic process (GO:0031146)' (18, 1.96, 0.001) 'negative regulation of TOR signaling (GO:0032007)' (22, 1.85, 0.001)
**Day 6**	'cellular response to interleukin-4 (GO:0071353)' (27, 1.54, 0) 'cellular senescence (GO:0090398)' (28, 1.51, 0) 'T cell homeostasis (GO:0043029)' (25, 1.48, 0.001) 'ER to Golgi vesicle-mediated transport (GO:0006888)' (60, 1.45, 0) 'anterograde synaptic vesicle transport (GO:0048490)' (16, 1.43, 0.038)	**Day 77**	'oxygen transport (GO:0015671)' (16, 1.36, 0.023) 'protein export from nucleus (GO:0006611)' (27, 1.35, 0) 'positive regulation of osteoclast differentiation (GO:0045672)' (22, 1.34, 0.001) 'cytolysis (GO:0019835)' (22, 1.34, 0) 'retrograde vesicle-mediated transport, Golgi to ER (GO:0006890)' (25, 1.31, 0.002)
**Day 10**	'negative regulation of oxidative stress-induced intrinsic apoptotic signaling pathway(GO:1902176)' (18, 1.43, 0.017) 'nucleosome disassembly (GO:0006337)' (17, 1.39, 0.011) 'protein-DNA complex disassembly (GO:0032986)' (17, 1.39, 0.011)'formation of translation preinitiation complex (GO:0001731)' (19, 1.38, 0.046) 'positive regulation of release of cytochrome c from mitochondria (GO:0090200)' (26, 1.37, 0.015)	**Day 98**	'cellular response to ammonium ion (GO:0071242)' (17, 1.87, 0) 'neurotransmitter secretion (GO:0007269)' (57, 1.78, 0) 'phosphatidylinositol acyl-chain remodeling (GO:0036149)' (16, 1.78, 0.004) 'glutamate secretion (GO:0014047)' (18, 1.78, 0.003) 'regulation of sensory perception of pain (GO:0051930)' (23, 1.78, 0)
**Day 14**	'positive regulation of protein dephosphorylation (GO:0035307)' (16,1.63, 0.013) 'DNA integration (GO:0015074)' (20, 1.63, 0.121) 'positive regulation of acute inflammatory response (GO:0002675)' (26, 1.49, 0.005) 'nucleotide-binding oligomerization domain containing signaling pathway (GO:0070423)'(28, 1.44, 0.025) 'cellular glucuronidation (GO:0052695)' (17, 1.40, 0.148)	**Day 119**	'modulation of growth of symbiont involved in interaction with host (GO:0044144)' (17, 1.41, 0.019) 'negative regulation of growth of symbiont in host (GO:0044130)' (17, 1.41, 0.019) 'regulation of growth of symbiont in host (GO:0044126)' (17, 1.41, 0.019) 'negative regulation of growth of symbiont involved in interaction with host (GO:0044146)' (17, 1.41, 0.019) 'chromatin silencing (GO:0006342)' (21, 1.37, 0.008)
**Day 35**	'Arp2/3 complex-mediated actin nucleation (GO:0034314)' (17, 1.87, 0.045) 'positive regulation of synaptic transmission, glutamatergic (GO:0051968)' (18, 1.78, 0.005) 'double-strand break repair via nonhomologous end joining (GO:0006303)' (18, 1.72, 0.065) 'non-recombinational repair (GO:0000726)' (18, 1.73, 0.065) 'immunoglobulin mediated immune response (GO:0016064)' (16, 1.72, 0.023)	**Day 140**	'regulation of peptidyl-serine phosphorylation of STAT protein (GO:0033139)' (18, 1.27, 0.079) 'positiveregulation of peptidyl-serine phosphorylation of STAT protein (GO:0033141)' (18, 1.27, 0.079) 'mitochondrial respiratory chain complex I assembly (GO:0032981)' (16, 1.24, 0.06) 'mitochondrial respiratory chain complex I biogenesis (GO:0097031)' (16, 1.24, 0.06) 'NADH dehydrogenase complex assembly (GO:0010257)' (16, 1.24, 0.06)

### 2.2 Biological process network to find perturbed paths in the network

**Fig 2:**
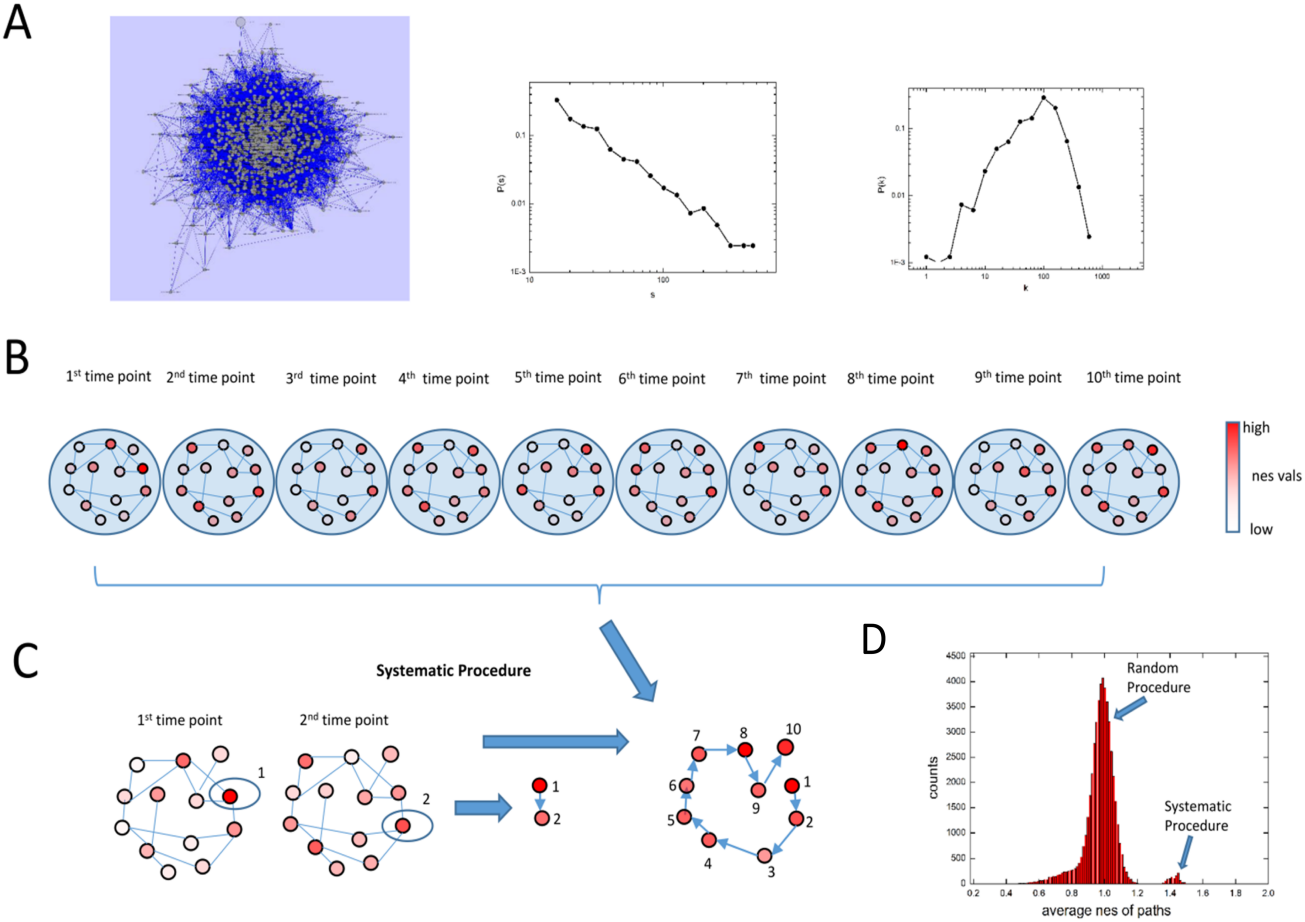
Biological Process network and finding perturbed paths in the network. (A) Network of connected biological processes is shown with 816 nodes and 51549 edges. Node size is proportional to number of genes in the corresponding process. In the middle inset, the distribution of number of processes (normalized to total number) with size within a bin is plotted against the starting of the bin s. In the rightmost inset, the distribution of number of processes (normalized to total number) with degree within a bin is plotted against the starting of the bin k. (B) A schematic is shown where overlaying the nes matrix on the network of biological processes gives 10 versions of the network. (C) Systematic procedure is shown where the circled process 1 is first selected having highest nes value among all processes at 1st time point. Then, the nes values of its connected processes at 2nd time point are checked and the one with highest nes value is selected as shown as circled process 2 in network at 2nd time point. This gives a path of 2 nodes going from process 1 to process 2. Repeating this process for further networks at 3rd, 4th etc. time point in (B) gives a path of 10 nodes. (D) The average nes of paths when generated randomly as well as when generated using systematic procedure shows that paths generated using systematic procedure has paths perturbed with high average nes values.

To find how processes perturbed at the initial time point affects processes perturbed at later time point, we considered looking at connectivity between biological processes. We thus made a network of these biological processes where nodes are biological processes and edges between two nodes signify that at least one gene is shared between the two biological processes. The network is shown in Fig 2A containing 816 nodes and 51549 edges. The distribution of number of genes present in each process is shown in Fig 2A middle panel which follows power law distribution suggesting few processes contain a large number of genes while a large number of processes contain few genes. We also checked the degree of each node and plotted its distribution in Fig 2A rightmost panel. Here also except few low and high degree process, most process were connected at a medium degree. Highest degree process here are 'coagulation (GO:0050817)' and 'blood coagulation (GO:0007596)' participating with 594 processes each. Here, it should be noted that while searching for paths in the network, these processes might come again and again due to their well connectivity. In that case, these processes would become most important. Now, overlaying the nes values of all 816 processes for a single time point on this network gives a network of processes with different levels of perturbation. Overlaying nes values of 816 processes at next time point generates same network with changed perturbed values. Thus, overlaying 10 vectors of nes values on this network generates 10 versions of this network with different perturbation values of the same processes. A schematic diagram is shown schematically in Fig 2B.

Next, we defined a path of length 10 made up of processes with high nes values at respective time points i.e. 1st process in the path have highest nes value at the 1st time point, 2nd process in the path have highest nes value at the 2nd time point and so on. Thus the obtained path will have the highest average nes value. To find such paths, we first used a brute force approach, i.e. randomly generating paths of length 10. A distribution of average nes values of 50000 such random paths is shown in Fig 2D. In the distribution, checking the paths with highest average nes values gave paths with values near to 1.2. To find paths with higher average nes values, we tried a systematic procedure outlined in Fig 2C. Here, we first selected the process having highest nes value at 1st time point as 1st process of the path and then checked the nes values of its interactors in the network at 2nd time point. Among the second interactors, we selected the interactor with highest nes value to be the 2nd process of the path. Next, we checked the nes values of interactors of the 2nd selected process in the 3rd time point and selected the interactor with highest nes value as the 3rd process of the path and so on. Repeating this for 10 time points gave a path of length 10. To find high perturbed paths we need to consider all processes in the analysis to find all possible paths and then select high perturbed paths. However, this would need high computational power. Here, for simplicity, we considered paths having process with next to maximum nes value instead of maximum nes value. In this procedure, at every instance of selection, we have two options i.e. selecting process with maximum nes value or next to maximum. Total 10 instance of selection with 2 options at each instance gives 2^10 = 1024 paths. The distribution of average nes values of these 1024 paths along with 50000 randomly sampled paths is shown in Fig 2D clearly showing that sampling with our systematic procedure gives higher average nes value which would be rare to obtain by random search.

We now want to establish whether the links between perturbed processes in the obtained paths are observed just because of high degree or have some functional significance. To test this, we computed the probability of getting these links in randomly shuffled networks of biological processes. We generated 10000 such random networks where links are shuffled and degree of each node is kept same. We then counted the number of networks in which we are getting the original observed link and divided the count by 10000 to get the probability for each observed link. Here, it should be noted that a link with low probability implies that the link is not due to the high degree of connected processes and thus might have some be functional importance. We plotted the distribution of link probabilities for all the links present in set of perturbed paths and also distribution of link probabilities present in the original network of processes (S4 Fig). We found a significantly low probability when links are taken from perturbed paths (mean p val = 0.23±0.20,) as compared to total links (mean p val = 0.45±0.30). The two distributions are significantly different with probability less than 0.0001 (by t-test and Mann-Whitney U-test). This gave us the confidence that the majority of links observed in our perturbed paths are functionally significant. Considering that the biological processes in the paths are temporally connected and since the connections are significant, these links might carry some causal information.

### 2.3 Significant overlap between set of perturbed paths

To study biologically such a large set of 1024 perturbed paths is challenging. We observed that some of the 1024 paths, converged at some time points. For example, as shown in Table 2, we found that two paths, say path a and path b, which start from two different processes at Day 1, follow different processes until Day 14 after which both the paths converge at same process. That is, according to our systematic procedure, both 'glutamine metabolic process' and 'translation' process have same connected process: 'positive regulation of synaptic transmission, glutamatergic' as the highest nes value or 2^nd^ highest nes value process at Day 35 among their respective connecting processes. This example implies that perturbed set of paths can converge or in other words overlap which we termed as overlap factor. We quantified this overlap factor for a specific path by below method.

**Table 2:** Two example paths which start from different processes and converge to same process at Day 35.

Time point	Path a	Path b
Day 1	'preassembly of GPI anchor in ER membrane (GO:0016254)'	'negative regulation of translational initiation (GO:0045947)'
Day 6	'mannosylation (GO:0097502)'	'nuclear-transcribed mRNA catabolic process, nonsense-mediated decay (GO:0000184)'
Day 10	'dolichol-linked oligosaccharide biosynthetic process (GO:0006488)'	'formation of translation preinitiation complex (GO:0001731)'
Day 14	'glutamine metabolic process (GO:0006541)'	'translation (GO:0006412)'
Day 35	'positive regulation of synaptic transmission, glutamatergic (GO:0051968)'	'positive regulation of synaptic transmission, glutamatergic (GO:0051968)'
Day 56	'glutamine metabolic process (GO:0006541)'	'glutamine metabolic process (GO:0006541)'
Day 77	'protein ADP-ribosylation (GO:0006471)'	'protein ADP-ribosylation (GO:0006471)'
Day 98	'substantia nigra development (GO:0021762)'	'substantia nigra development (GO:0021762)'
Day 119	'chromatin silencing (GO:0006342)'	'chromatin silencing (GO:0006342)'
Day 140	'cellular response to glucose starvation (GO:0042149)'	'cellular response to glucose starvation (GO:0042149)'

Denoting an *ith* path (*i = 1*:*M*, *M* = total paths) as a set of processes $p_{i}^{j}$ (j = 1:10) and set of edges $e_{i}^{j,j+1}$ (*j* = 1:9), where *j* indicates time at which the process is perturbed and superscript (*j*,*j+1*) on an edge indicates the edges connecting process $p_{i}^{j}$ and $p_{i}^{j+1}$. The edges also denote the genes which are present in both of its connecting processes.

$$P_{i}={\left\{p_{i}^{1},e_{i}^{1,2},p_{i}^{2},\ldots \ldots \ldots .p_{i}^{9},e_{i}^{9,10},p_{i}^{10}\right\}}^{},\overset{}{}i=1:M$$

Define overlap factor $O(e_{i}^{j,j+1})$ for an edge $e_{i}^{j,j+1}$ as a set which consists of paths such that each path in the set has same edge as that of query edge $e_{i}^{j,j+1}$

$$O(e_{i}^{j,j+1})={\left\{P_{m}|e_{m}^{j,j+1}=e_{i}^{j,j+1}\right\}}^{}{,}^{}m\leq M{,}^{}m\neq i^{}{,}^{}j=1:9^{}{,}^{}i=1:M$$

Define overlap factor *O*(*P_i_*) for a path *P_i_* as the union of paths present in overlap factors of its edges.

$$O(P_{i}^{})=\cup O{\left(e_{i}^{j,j+1}\right)}^{}{,}^{}j=1:9^{}{,}^{}i=1:M$$

**Fig 3:**
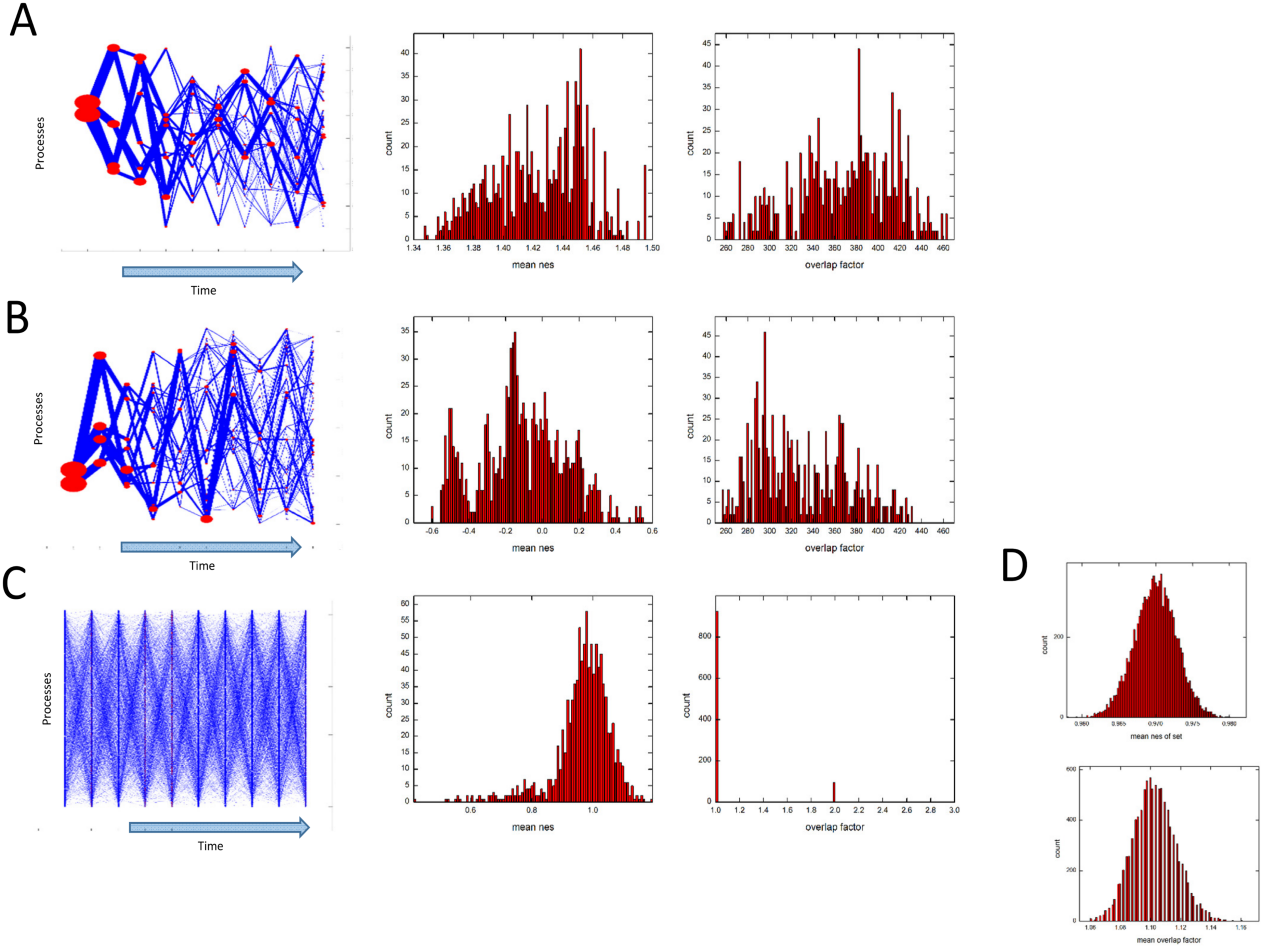
Significant overlap between set of perturbed paths. (A,B,C) In left panels, the visual display of set of 1024 paths are shown with edge thickness proportional to the overlap factor of corresponding edge and node size proportional to number of paths with same node. In middle panels, the distribution of mean nes value of 1024 paths is shown and in right panels, the distribution of overlap factor of 1024 paths is shown. For the set of 1024 perturbed paths in A, the overlap between paths can clearly be seen (left panel) with distribution of mean nes values of paths around 1.42 nes values. (Distribution of mean nes values is same as that shown in Fig 2C systematic procedure). Compare this with distribution of mean nes values of set of unperturbed paths in (B) middle panel showing higher nes values of perturbed paths as expected. The overlap factor of perturbed paths is around 380 (A, right panel) while that for unperturbed paths is around 340 (B, right panel) and this difference is statistically significant. This difference of overlap factors is also seen in visual display of paths in (A,B) left panels with thicker edges in plots of perturbed paths. Compare the overlap factors with that for random paths with no or very little overlap as seen in distribution (C, right panel) and also seen in visualization (C, left panel). (D) The mean of the distribution of mean nes values of 1024 randomly selected paths were calculated and this procedure repeated for 10000 sets of 1024 paths and this distribution is plotted in (D, upper panel). The mean nes values of set is low as also seen in distribution of mean nes values of a single set of 1024 randomly selected paths in (C, middle panel). Similar procedure was applied for overlap factors showing that for many sets of 1024 randomly selected paths, the mean of overlap factors of set remains around 1 as seen for a single set of 1024 randomly selected paths in (C, right panel).

The overlap between paths is visualized by plots in Fig 3 where x axis is time point, y axis is the process. In this plot, each path is showed by plotting its processes against their respective times and connecting them. We added thickness to these lines based on size of overlap factor sets of respective edges. We used specific statistical tests to assign significance to the obtained overlap factors. Firstly, we compared whether such overlap factors could be obtained using randomly chosen paths. For this, we took a set of 1024 randomly picked paths and calculated the overlap factor of each path and took the mean overlap factor. We also calculated the mean nes values of these paths. We repeated this procedure 1000 times to get a distribution of mean overlap factor and of mean nes values (Fig 3D). By comparing this distribution with actual overlap factor values for perturbed paths (Fig 3A), we found that mean overlap factor values 368.58 and mean nes values 1.42 of perturbed paths are significantly higher than for random paths nes = 0.97+-0.0029, overlap factor = 1.1025+-0.0145. Next, we wanted to check whether our results are due to an artifact of our systematic procedure. That is whether the overlap is due to the fact that we are always starting from one of the two highest nes value processes to get set of 1024 processes or is it a property specific to set of perturbed paths. Hence, instead of taking highest nes values in our systematic procedure, we took lowest nes value processes for our analysis and generated 1024 unperturbed paths keeping all the other steps of systematic procedure same. We found that the nes values and overlap factor of 1024 unperturbed paths are significantly low compared to that of perturbed paths (Fig 3B), the distributions of overlap factor for perturbed and unperturbed paths are different significantly with p value from Mann-Whitney U-test <0.0001. This proves that our results are not an artifact of our systematic procedure and are property specific to set of perturbed paths.

Having established this property, we used it to select a subset of paths from the set of 1024 paths which overlap with most of the other paths. We found that the path with highest overlap factor set contained 464 paths out of total 1024. To increase the percentage, we tried different combinations of two paths and found that there are four pairs of paths that had 822 paths in their overlap factor sets i.e. approx. 80% of total paths. These paths are mentioned in Table 3, where the processes at each time point are showed along with their respective genes perturbed at least 2 fold.

**Table 3:** Two paths are shown in path 1 column with same processes till Day 119 and two different processes at Day 140. Same for Path2. Two paths in Path1 and in Path2 pair up to give 4 pairs of paths representing 80% of all paths.

Time point	Path1		Path2	
	Process Name	Gene perturbed, abs fold change	Process Name	Gene perturbed, abs fold change
Day 1	preassembly of GPI anchor in ER membrane.	PIGC, ~32 fold	'negative regulation of translational initiation (GO:0045947)'	'EIF2AK1', 'RPL13A', >5 fold
Day 6	GPI anchor biosynthetic process	'PIGV', ~4 fold	'nuclear-transcribed mRNA catabolic process, nonsense-mediated decay '	'DCP1A', 'PARN', 'RPL31', 'RPS15A', > 2fold
Day 10	dolichol-linked oligosaccharide biosynthetic process	'ALG5', 'MVD', 'PMM1', 'SRD5A3', >2fold	'I-kappaB kinase/NF-kappaB signaling '	'NLRC3', 'UBA52', > 2 fold
Day 14	glutamine metabolic process	'ASNS', 'CAD', 'MECP2', 'PFAS', 'PHGDH', >2 fold	'nucleotide-binding oligomerization domain containing signaling pathway '	'CARD9', 'CHUK', 'MAP2K6', 'TNFAIP3', 'UBA52', >2 fold
Day 35	positive regulation of synaptic transmission, glutamatergic	'ADCYAP1', 'GLUL', 'NLGN2', 'NLGN3', 'NRXN1', 'NTRK1', 'NTRK2', 'OXTR', 'PTK2B', 'SHANK3', 'TNR', >2fold	'innate immune response in mucosa	‘CAMP', 'DEFA1', 'DEFB1', 'HIST1H2BC', 'HIST1H2BK', 'LTF', 'RPL39', > 2 fold
Day 56	positive regulation of blood vessel endothelial cell migration	'AKT1', 'ANGPT1', 'FGF2', 'HDAC9', 'HSPB1', 'PDGFB', 'PRKD1', 'PTGS2', 'THBS1', >2 fold	'modulation of growth of symbiont involved in interaction with host '	'CAMP', 'IFNG', 'LBP', 'LTA', 'MPO', > 2 fold
Day 77	positive regulation of peptidyl-threonine phosphorylation	'CHI3L1', >6 fold	'positive regulation of osteoclast differentiation '	'FOS', >25 fold
Day 98	positive regulation of protein dephosphorylation	'ADORA1', 'CALM1', 'DUSP26', 'PPP2R5A', 'PRKCD', >2 fold	'bicarbonate transport '	'AQP1', 'SLC4A10', 'SLC4A3', 'SLC4A4', 'SLC4A7', 'SLC4A8', 'SLC4A9', > 2 fold
Day 119	lymph node development	'CD248', 'CXCL13', 'CXCR5', 'FADD', 'IL15', 'LTB', 'NKX2-3', 'RORC', >2 fold	'hydrogen peroxide catabolic process '	'APOA4', 'CAT', 'MPO', 'TPO', >2 fold
Day 140	lung alveolus development/myeloid dendritic cell differentiation	'SFTPD, >4 fold/ 'CAMK4', 'UBD', >2 fold	'response to increased oxygen levels (GO:0036296)'/'response to hyperoxia '	CDKN1A', 'FAS', 'NCF2', > 4 fold

**Fig 4:**
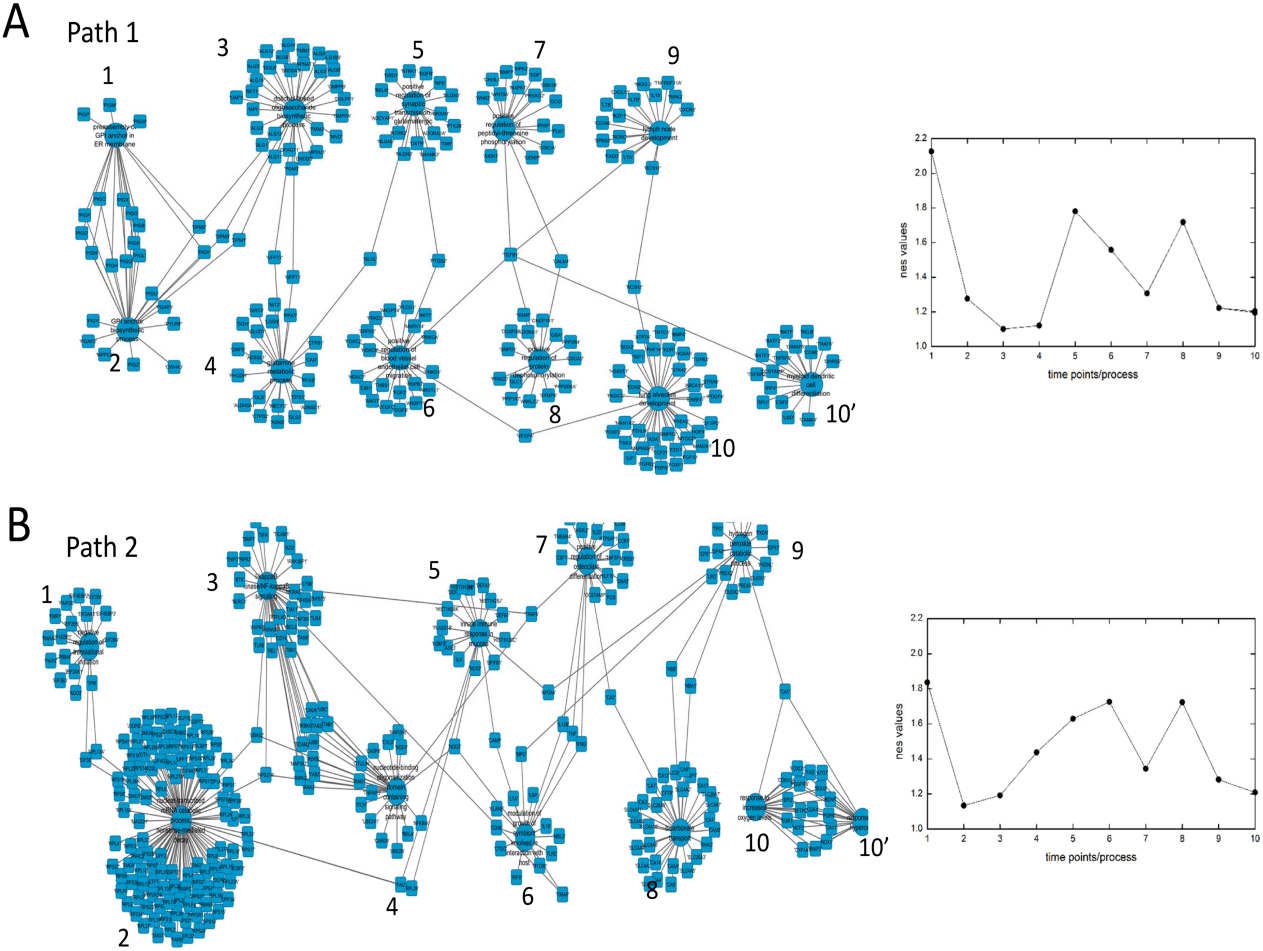
Paths overlapping with 80% paths. (A,B) Paths are shown as a network with two kinds of node: one representing processes and other genes. Nodes representing processes connects to the genes present in those processes. Numbers present near each process represent its position in the corresponding path and hence the time point. 10 and 10’ implies two different processes and implies that there are two paths with same processes at 1 to 9 time points and two different processes at 10th time point (A) Path1 representing two paths is shown along with the nes values of the processes at corresponding time points in the adjacent graph. The nes values for two processes at 10th time point are same and hence can’t be visually distinguished in graph. (B) Path2 representing two paths is shown along with the nes values of the processes at corresponding time points in the adjacent graph. The nes values for two processes at 10th time point are same and hence can’t be visually distinguished in graph.

To better depict the paths and capture the connectivity between processes, we made a gene process network where processes at consecutive time points are connected through common genes, see Fig 4. This connected network helped us to identify genes which are connecting processes at consecutive time points. For example, in path 1 shown in Fig 4A, we observe that genes like PIGC, PIGX and others connect processes ‘preassembly of GPI anchor in ER membrane’ perturbed at Day 1 with ‘GPI anchor biosynthetic process’ perturbed at Day 6. We also observed that some genes connect processes perturbed at more than 2 consecutive time points. For example, genes DPM2 and PIGV links process ‘preassembly of GPI anchor in ER membrane’ perturbed at Day 1 with ‘GPI anchor biosynthetic process’ perturbed at Day 6 and also ‘dolichol-linked oligosaccharide biosynthetic process’ perturbed at Day 10. Similarly we can extract genes linking processes at other time points and also for other paths. Thus the processes with their connecting genes forms the path which is capturing disease progression.

### 2.4 Precision of the selected paths

**Fig 5:**
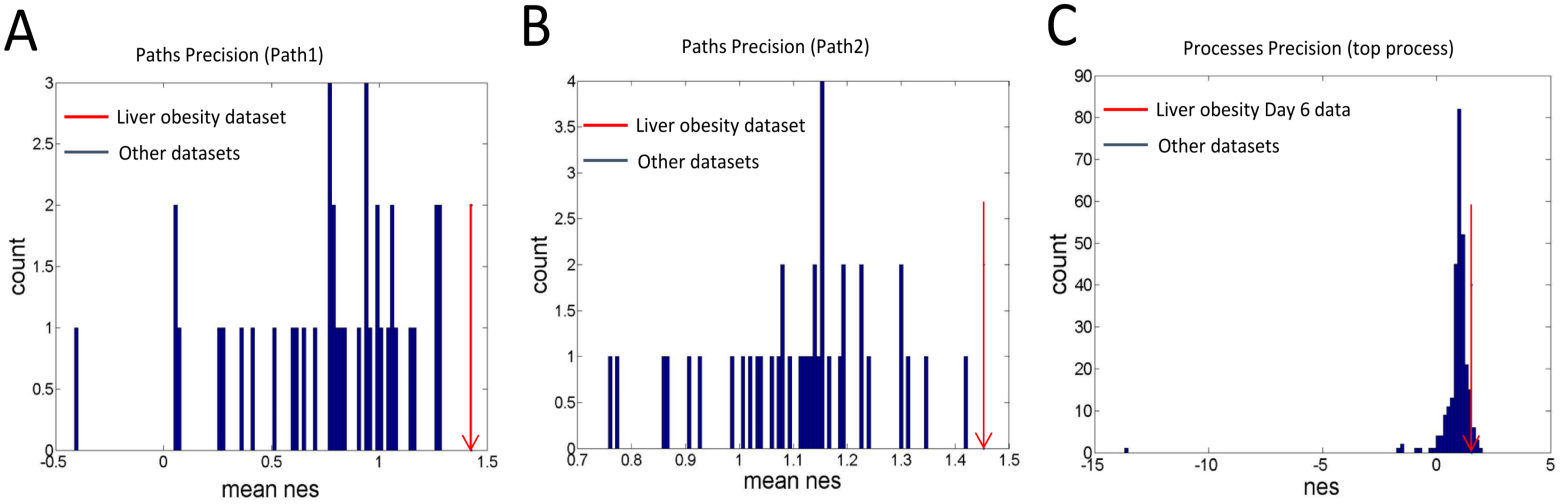
Precision of the selected paths. (A,B) Distribution of mean nes values of a path in Path1 column and a path in Path2 column from Table 3 showing that the obtained paths have high mean nes values when obtained from liver obesity dataset as compared to when obtained from other temporal datasets showing a 100% precision. (C) Distribution of nes values of topmost perturbed process at Day 6 (Table 1) from liver obesity dataset as compared to when obtained from other temporal datasets showing some datasets give nes values of the given process more than that as obtained from liver obesity dataset giving a precision of 9%.

Having established the paths which are capturing the disease progression, we now checked the precision of the selected paths. That is we wanted to check how specific are the obtained paths with respect to the particular dataset representing a particular tissue in a disease state. For this, we used temporal datasets from NCBI repository under GEO accession number GSE63178. The database consisted of 40 temporal datasets, one of which was used in our study to find paths and other 39 datasets were used to calculate the precision of selected paths. The database consisted of microarray data obtained from temporal gene expression profiling of multiple murine tissues with different diet regimens (Table 4) [15]. We used the paths as given in Table 3 for our analysis in all 39 temporal datasets. We calculated the nes values of the processes of the selected path and obtained the mean nes value for the same path in all 39 datasets. Some temporal datasets didn’t have all the 10 time points as of the liver dataset. Some of them had only last few time points. In such cases, we used only the corresponding last few processes of the path to calculate nes values of process and average nes value of path. We plotted the average nes values for a path corresponding to each dataset in Fig 5A and 5B. We observed that the mean nes value of path in liver obesity dataset is higher among all other datasets. This means the obtained paths are perturbed specifically in the liver obesity dataset and are highly precise to the source dataset.

**Table 4:** Datasets used in the precision analysis.

No.	Murine tissues under low-fat control diet (LFC), high-fat diet (HFC), and three different doses (5 μg, 20μg and 75 μg) of ethano-botanical formulation Kal-1 with HFC diet. (GSE63178)
1	brown adipose (GSE63168)
2	brown infiltrating macrophages (GSE63169)
3	epididymal adipose (GSE63170)
4	epididymal infiltrating macrophages (GSE63171)
5	subcutaneous adipose (GSE63172)
6	subcutaneous infiltrating macrophages (GSE63173)
7	Hippocampus (GSE63174)
8	Liver (GSE63175)
9	skeletal muscle (GSE63176)
10	Spleen (GSE63177)

To compare this precision with the scenario when processes from individual time points are used, we took the topmost process from Day 6 (Table 1). We calculated the nes value of this process in other time points of this dataset and then in other datasets giving a total of 194 nes values. The distribution of these values are shown in Fig 5C showing an overlap of nes values from liver obesity Day 6 dataset and nes values from other time points and other datasets. We found that 10 datasets had nes values of this process higher than liver obesity Day 6 dataset. Thus predicting the source dataset using process would give a false positive prediction and hence a precision of 9% (with 10 false positives and 1 true positive using a threshold nes value just lower than that of selected process in liver obesity Day 6 dataset, compared to paths case with 0 false positive and 1 true positive prediction using a threshold just lower than mean nes value of path in liver obesity dataset). From a statistical point of view, this would mean that the probability to obtain from another dataset a mean nes value at least as high as the one obtained from the liver obesity dataset would be 0.052 (= 10/194).

This proves that processes are not specific to source dataset while the obtained paths are specific to source dataset.

## 3 Discussion

Finding links from a biological data is a desirable aim and can help in mechanistic understanding of underlying processes at work. Time series microarray data can be used to find such links as well as giving temporal direction to these links. In the present study, using a time series microarray data, we aimed to capture the disease progression at process and gene level. For this, we constructed a biological process network. Networks between biological processes or more generally sets of genes have also been used in other studies linking biologically relevant terms [12, 16] to form networks and obtaining important biological insights. However, temporal information was not present in such networks which are very important to capture disease progression. To fill that gap, we have overlaid the temporal information from our data on our constructed biological process network resulting in a temporal process network. We then used this temporal process network to link the processes perturbed at consecutive time points. This resulted in paths that link initial perturbed processes with late perturbed processes and helped us to capture the disease progression. For example, we observed in path 1 (Fig 4A) that signaling processes which are perturbed at Day 1 are linked to glutamate metabolic related process through enzymes GFPT1 and GFPT2 which is further linked to synaptic transmission and cell migration related processes through enzyme GLUL. This is finally connected to inflammatory related processes at later time points through protein TGFB1. Inflammatory related processes have been observed in obesity condition [17] and our analysis can give the initial set of processes potentially leading to these processes. In another example, we observed in path 2 (Fig 4B), ‘nuclear transcribed mRNA catabolic process, nonsense mediated decay’ is perturbed initially which is linked to 'I-kappaB kinase/NF-kappaB signaling ' processes through gene UBA52 which is then connected to 'innate immune response in mucosa’ through gene NOD2. This process is then connected to 'hydrogen peroxide catabolic process through the gene APOA4.

We used t-test and other tests to provide meaning to the captured links present in the paths. Our statistical tests gave us confidence that the observed links are just not because of degree factor of processes. Thus we hypothesized that given two processes are perturbed at consecutive time points and are connected, there is a high chance that perturbation of a process at a time point might cause perturbation of other process at the next time point through these common genes. Although, this is a result drawn from a statistical testing and the ultimate validation can only be done through experiments. However, despite this limitation, we believe that our framework can help in generating hypothesis of the causal mechanisms behind observation of different perturbed processes at different times of the experiment.

As final validation of the captured paths, we showed that the selected paths are more precise than biological processes in defining the biological state. We must mention here that as the datasets used in precision analysis were taken from different tissues as well as from different variations of high fat high sugar diet condition (Table 4). Due to similar conditions as our liver obesity dataset, one would expect that the paths would have similar average nes values. Our results of high average nes values of the paths in liver obesity than in other datasets in spite of the highly similar conditions of the datasets suggests that the paths are highly precise in capturing the conditions of our dataset.

Thus, in conclusion we can say that our analysis helped us to extract genes that can be hypothesized to play an important role in disease progression which can be experimentally validated. One can further take up these genes for mechanistic understanding of disease progression. Finally, the procedure we have presented here can be used with any time series microarray, proteomics data to find links between processes perturbed at different time points.

## 4 Material and methods

### 4.1 Processing of microarray data

The microarray data was obtained from an experiment where one group of mice were fed with high fat high sucrose diet (treated group) and another group with normal diet (control group) for certain days before taking tissue samples from both groups of mice. Both groups of mice were fed respective diets for following days: Day1, Day 6, Day 10, Day 14, Week 0, Week 3, Week 6, Week 9, Week 12, Week 15 and Week 18. This experiment was repeated for three times. Then, microarray experiment was performed on tissue samples and after suitable normalization of the signal intensities of each probe using Agilent Genespring GX software, three values of log fold change for control sample and treated sample were obtained for each probe and at each time for each tissue. Further details of the experiment are given in [15].

This data for liver tissue was downloaded from the NCBI repository under GEO accession number GSE63175. The data also contains information about data where mice were fed with high fat high sucrose diet plus an ayurvedic formulation which is out of scope from our present study. These data correspond to columns with columns header “P2_HFx_y “(x: 5,20,75 and y contains time point and sample number information) and were removed. The column headers have information of time point of experiment in days as well as weeks. Weeks were recorded in experiment as number of weeks after Day 14. Thus, 14 days were added while converting weeks to days. This implies Day 14 and Week 0 would correspond to same time i.e. experiment done twice; thus Day14 and Week 0 information was combined after making the final matrices as mentioned at end of this section. This resulted in final time points as: Day 1, Day6, Day 10, Day 14, Day 35, Day 56, Day 77, Day 98, Day 119, and Day 140 as reported in this study. The steps used in Processing of Microarray data are described below and shown in a schematic diagram in S1 Fig.

For each probe, the mean of log fold change for treated samples were calculated and a pvalue signifying difference between three control values and three treated values was generated by using t-test. The data contains 40628 probes which correspond to 29411 gene symbols. Gene symbol information for each probe was taken from column with column header “Gene symbol”. Here, multiple probes correspond to same genes.

Step 1. First, we filtered the data to have only those genes that are changed for at least 2 fold in all three treated samples at a time point. In case two probes corresponding to same gene satisfy this condition, the probe with minimum p value was chosen. We repeated this process for data at different time points and combined all filtered genes together to form a matrix with filtered genes and time points with fold change values of all filtered genes inserted at respective time points. In the matrix, there would be many genes with no fold change values at some time points. For these genes, we used the following steps to insert values at these time points (i.e. where these are not >2 fold changed).

Step 2.For the genes with missing values, we went back and searched their probe’s fold change values and in case if we find any two values out of three are outside the interval -1 and 1, we selected those probes and go to step 3. If no probe of the selected gene satisfies above criteria, we then selected those probes which would have values at three samples within -1 and 1 and go to Step 3.

Step 3. The selected probe’s average over three samples were taken if in all three cases the value is greater/less than 0. If multiple probes of a gene satisfied this condition, probe with minimum p value was chosen. If no probe out of selected probes satisfy this condition, probe’s average value over two (out of three) samples with value greater/less than 0 was taken. For multiple probes satisfying this condition, probe with minimum p value was taken. For the probe chosen, if the average value was less than 0.8/greater than -0.8, a dummy number 0.001 was inserted, else the average value was inserted in the matrix.

The resulting matrix contained log fold change values at eleven time points. We combined Day 14 and Week 0 information in following way. If defined perturbed gene as having absolute log fold change >1 and negative (positive) perturbed gene as having log fold change <-1 (>1) at a time point. Now, if a gene’s value in both time points are perturbed in same direction (-ve or +ve), we took average values as merged value. If values are perturbed in opposite direction in two time points, we assigned a dummy number (as used above) to that gene as merged number. If gene’s value is perturbed in only in 1 time point, we used that value as merged value and if value is not perturbed in both time point, we assigned one of the non perturbed value as merged value. Now, we checked, in resulting matrix of 10 time points, if a gene is not perturbed in even a single time point, we removed those genes.

The resulting matrix contained log fold change values at ten time points for 19303 genes. The matrix was clustered using default clustergram function of matlab which uses algorithm of Eisen et al. (Eisen, et al., 1998) resulting in heatmap shown in Fig 1A.

### 4.2 Gene Ontology Biological Process list

The Gene Ontology Biological Process list was obtained from Enrchr Library [7] section under the name ‘GO_Biological_Process’ containing 5192 processes each signifying a set with specific set of genes together totaling 14264 genes. From this list of 5192 processes, we made a list of 816 processes using following method. We included a process in our list only if no other process was a subset of this process. The list of 816 biological process names is provided in S1 Table. We then applied a cutoff such that all processes in our list contained more than 15 genes.

### 4.3 Calculation of es, nes and pvalues

We followed a method as described in [8] to calculate es, nes and pvalues. Briefly, for a list of N genes ranked in descending order according to their absolute log fold change values (for a gene) say at time t and for a process say S containing total genes, the following terms are calculated. The fraction of genes in S (‘hits’) weighted by their fold change and fraction of genes not in S (‘miss’) present upto a given position i are calculated as given in formulas below.

$$P_{hit}(S,i)=\sum_{\begin{array}{l} g_{j}\in S\\ j\leq i \end{array}}\frac{\left|r_{j}\right|}{N_{R}}$$

where $N_{R}=\sum_{\begin{array}{l} g_{j}\in S \end{array}}\left|r_{j}\right|$

$$P_{miss}(S,i)=\sum_{\begin{array}{l} g_{j}\notin S\\ j\leq i \end{array}}\frac{1}{\left(N_{}-N_{H}\right)}$$

Here, r_j_ is the fold change of gene j, N is total number of genes, N_H_ is the number of genes in S. Enrichment score (es) is calculated as maximum plus minimum of *P_hit_* − *P_miss_*. The plot of *P_hit_* − *P_miss_* for a particular process and for fold change values of genes at Day 1 is shown in Fig 1B. Normalized enrichment score was calculated by calculating es for each of 1000 random permutations of N gene labels and getting a distribution of es scores. Pvalues are calculed as the fraction of number of times es obtained is greater/less (according to sign of es as positive/negative) than or equal to actual es score. Normalized enrichment score is calculated by dividing the actual es score by the mean of positive/negative (according to sign of actual es) portion of the null distribution.

## Supporting information

S1 Fig Schematic diagram illustrating the steps used in processing of microarray data. For each gene (represented by one/many probes), its fold change value (FC_1_, log_2_ transformed) at each time point is calculated as in step 1. For the genes where condition of step 1 is not satisfied at some time points (as represented by empty boxes in the matrix), step 2 and step 3 are followed to get the fold change values (FC_2_) and inserted at respective places in the matrix. Then time points t4 and t5 are combined as mentioned in text to give Final Matrix.
(PDF)

S2 Fig Gene set enrichment analysis to find perturbed biological processes. An example of gene set enrichment analysis method is shown for a process receiving high nes value at Day 1. Most instances of genes of this set are present towards left which results in high es and nes value and signifies that most genes of this process are perturbed.
(PDF)

S3 Fig High nes values imply significantly perturbed process and high mean absolute fold change values of its genes. (A) For all the processes the nes values calculated at each time point were plotted against the corresponding–log10 pvalue and shows that as nes values of a process increases the corresponding–log10(pvalue) also increases signifying that process with high nes values are significantly perturbed. (B) Here, for each process, the average absolute fold change of its genes at each time point is calculated and this value is plotted against the nes values of these processes. The plot shows as the nes values of a process increases, the average absolute fold change values of its genes also increases.
(PDF)

S4 Fig Probability of edges. Probability of obtaining given edges by chance is plotted for all edges as well as edges from set of perturbed paths and clearly shows that probabilities are low for edges from set of perturbed paths as compared to total edges.
(PDF)

S1 Table Biological process names. The list of 816 biological process names.
(XLS)
