# Supplementary figures and images for: Tracking disease progression by searching paths in a temporal network of biological processes
Source: PLoS One. 2017 Apr 27;12(4):e0176172. doi: 10.1371/journal.pone.0176172 (PMC5407620; doi:10.1371/journal.pone.0176172)

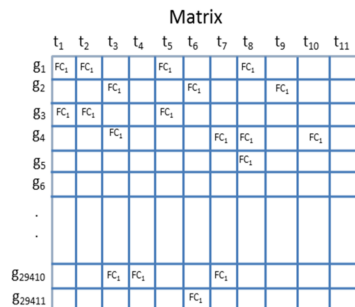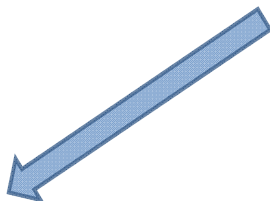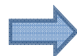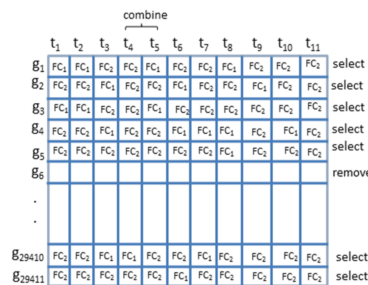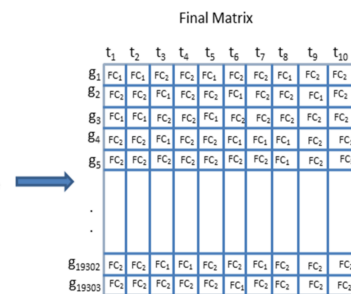

Supplement: S1 Fig — For each gene (represented by one/many probes), its fold change value (FC1, log2 transformed) at each time point is calculated as in step 1. For the genes where condition of step 1 is not satisfied at some time points (as represented by empty boxes in the matrix), step 2 and step 3 are followed to get the fold change values (FC2) and inserted at respective places in the matrix. Then time points t4 and t5 are combined as mentioned in text to give Final Matrix. (PDF) [file pone.0176172.s001.pdf]

transcription elongation from RNA polymerase III promoter  
(GO:0006385), t=Day1, genes=11, es=0.8359, nes=1.806, pval=0.002

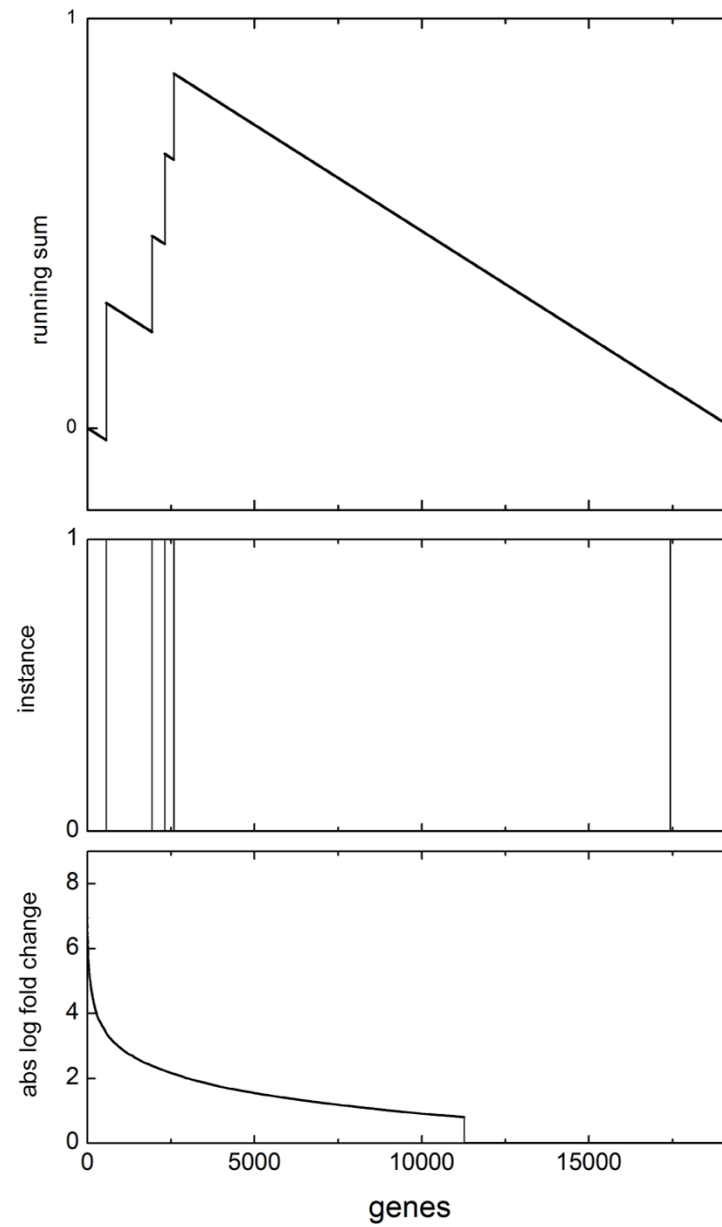

Supplement: S2 Fig — An example of gene set enrichment analysis method is shown for a process receiving high nes value at Day 1. Most instances of genes of this set are present towards left which results in high es and nes value and signifies that most genes of this process are perturbed. (PDF) [file pone.0176172.s002.pdf]

A

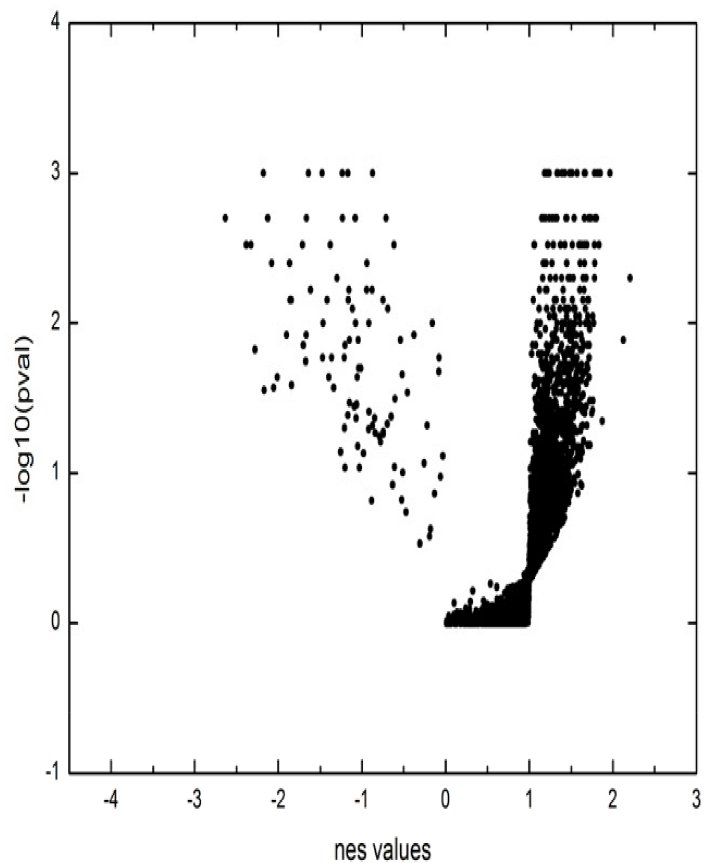

B

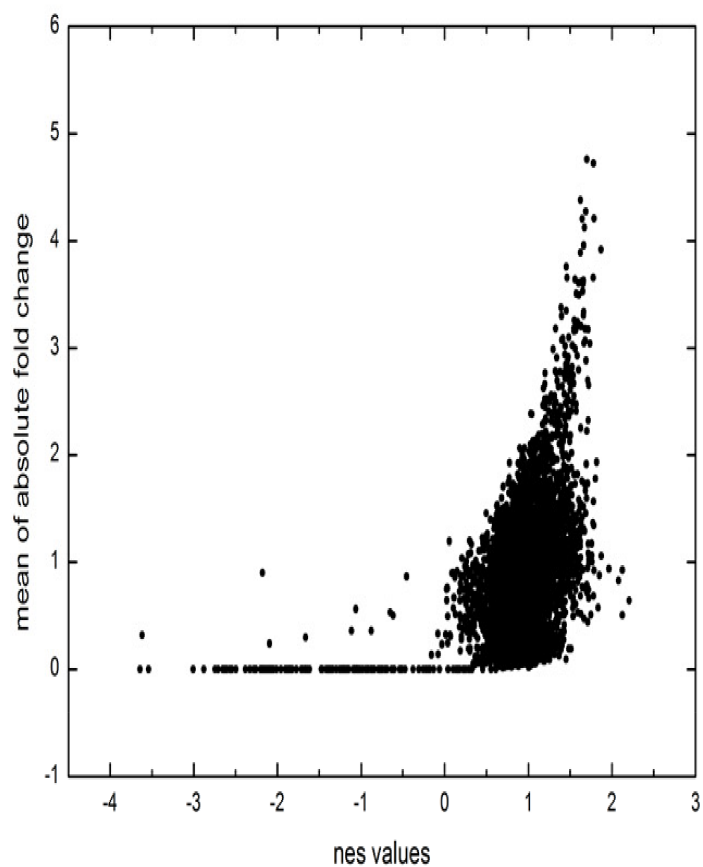

Supplement: S3 Fig — (A) For all the processes the nes values calculated at each time point were plotted against the corresponding–log10 pvalue and shows that as nes values of a process increases the corresponding–log10(pvalue) also increases signifying that process with high nes values are significantly perturbed. (B) Here, for each process, the average absolute fold change of its genes at each time point is calculated and this value is plotted against the nes values of these processes. The plot shows as the nes values of a process increases, the average absolute fold change values of its genes also increases. (PDF) [file pone.0176172.s003.pdf]

Total edges

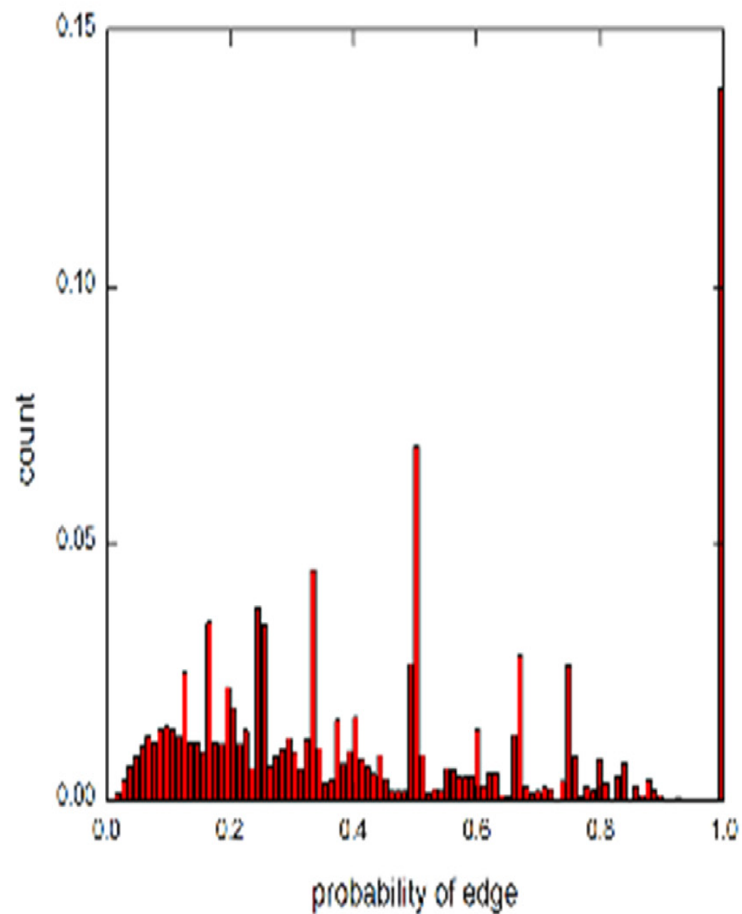

Edges from perturbed paths

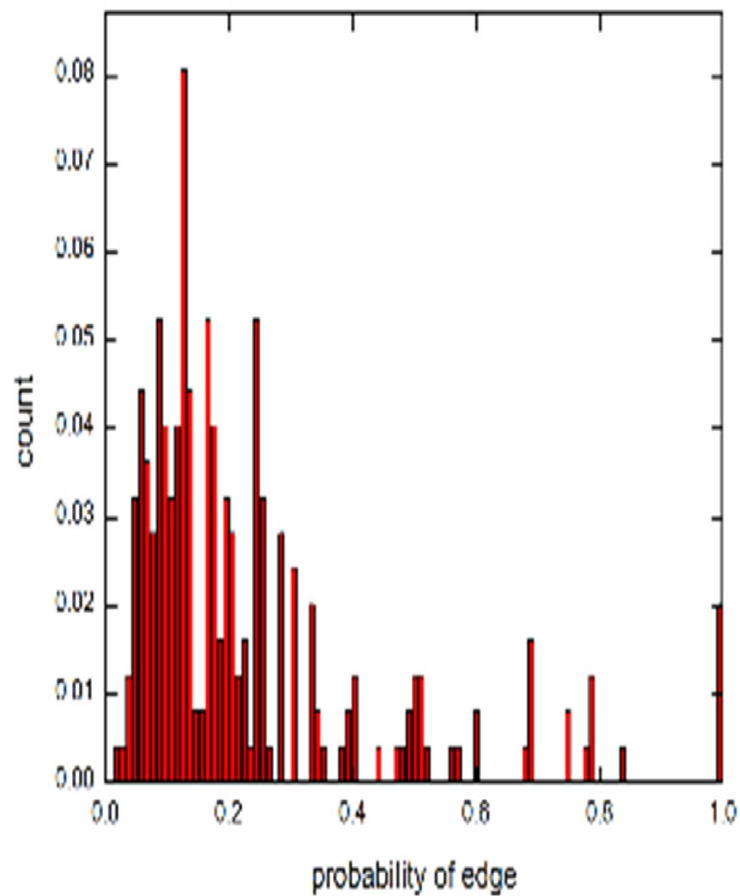

Supplement: S4 Fig — Probability of obtaining given edges by chance is plotted for all edges as well as edges from set of perturbed paths and clearly shows that probabilities are low for edges from set of perturbed paths as compared to total edges. (PDF) [file pone.0176172.s004.pdf]
